# Changes in Metabolites from Bovine Milk with β-Casein Variants Revealed by Metabolomics

**DOI:** 10.3390/ani10060954

**Published:** 2020-05-30

**Authors:** Zhongwang Lv, Hui Liu, Yongxin Yang, Dengpan Bu, Changjiang Zang, Kailun Yang, Xiong Yu, Jiaqi Wang

**Affiliations:** 1 College of Animal Science, Xinjiang Agricultural University, Urumqi 830052, China; lvzhongwang@sina.com (Z.L.); liuhui2001@163.com (H.L.); zcj780@126.com (C.Z.); yangkailun2002@aliyun.com (K.Y.); yuxiong8763601@126.com (X.Y.); 2 State Key Laboratory of Animal Nutrition, Institute of Animal Science, Chinese Academy of Agricultural Sciences, Beijing 100193, China; yyongxin@yahoo.com (Y.Y.); budengpan@caas.cn (D.B.); 3 Anhui Key Laboratory of Livestock and Poultry Product Safety Engineering, Institute of Animal Science and Veterinary Medicine, Anhui Academy of Agricultural Sciences, Hefei 230031, China

**Keywords:** β-casein variant, bovine milk, metabolite, metabolomics

## Abstract

**Simple Summary:**

Changes in milk protein content have been associated with β-casein variants. However, the specific changes in the metabolites of β-casein variant milk remain unclear. Thus, a metabolomics approach was employed to determine the abundance of different metabolites in milk samples with β-casein variant A1/A1, A2/A2, and their heterozygote. The metabolites with the highest abundance were methionine, proline, and α-lactose in variant A2/A2 milk, choline, glycine, citric acid, and cyclic adenosine monophosphate (cAMP) in variant A1/A1 milk, and uric acid and cytosine in heterozygote milk. These results may facilitate further explorations of the differences in the biosynthesis of milk components in the mammary gland and help to elucidate the potential influence of β-casein variants on the physiological function of milk.

**Abstract:**

β-casein is a primary protein in milk, and its variants have been associated with changes in the protein content of bovine milk. However, there has been little research focused on the effects of β-casein variants on milk metabolites. In the present study, dairy cows producing milk with β-casein variant A1/A1 (A1), A2/A2 (A2), and their heterozygote A1/A2 (A12) were screened by a high-resolution melting method. Individual milk samples were then collected from each of the cows, and the milk metabolites were separated and analyzed using nuclear magnetic resonance spectroscopy- and liquid-chromatography mass spectrometry-based metabolomics techniques. Differences in metabolites among the variant groups were evaluated by multivariate statistical analysis. The relative abundances of methionine, proline, and α-lactose were the highest in β-casein variant A2 milk, whereas choline, glycine, citric acid, and cyclic adenosine monophosphate (cAMP) showed the highest abundances in variant A1 milk. Metabolic pathways analysis indicated that the differential metabolites between variants A1 and A2 were involved in pantothenate and coenzyme A biosynthesis, butanoate metabolism, and valine, leucine, and isoleucine biosynthesis. Our results reveal the differences in milk metabolites among the β-casein variants A1, A2, and the heterozygote. These findings, thus, provide novel insights into the effects of β-casein variants on milk metabolites, facilitating further research into the mechanism of the biosynthesis of milk components in the mammary gland and the potential physiological function of milk associated with β-casein variants.

## 1. Introduction

β-casein is a major milk protein, comprising approximately 30% of total milk proteins, and participates in the formation of the casein micelle, which prevents the aggregation of a wide range of proteins [1,2]. Multiple variant forms of β-casein have been identified in bovine milk [3,4], including β-casein variant A1/A1 (A1) and variant A2/A2 (A2) as the predominant forms, in which the 67th amino acid residue is histidine and proline, respectively [5], and their phenotypes in dairy cow milk have been identified [6,7]. Several studies have shown the association of β-casein variants with several milk components (e.g., α_S1_-casein, β-casein, and β-lactoglobulin) [8,9] and their contributions to the structural differences in the casein micelle and molecular chaperone activity [2]. More recently, we performed a proteomics analysis of casein micelle, whey, and milk fat globule membrane protein components among β-casein variants A1, A2, and their heterozygote A1/A2 (A12) in dairy cows, revealing the distinct proteins among β-casein variants milk. Specifically, ceruloplasmin, protein S100-A9, and cathelicidin-2 had the highest abundance in variant A1, whereas antithrombin-III, protein S100-A8, and protein S100-A12 had the highest abundance in variant A2 (unpublished data). Moreover, milk with bovine β-casein variants A2 and A1 was reported to induce the formation of a yogurt gel microstructure [10]. Collectively, these findings indicate that differences in milk protein components could be related to β-casein variants.

Milk also contains a complex mixture of metabolites, such as amino acids, carbohydrates, lipids, vitamins, and nucleotides [11]. Because the milk metabolite composition is closely correlated to its nutritional significance and involved in multiple healthy benefits [12], several previous studies have investigated the changes in milk metabolites among dairy animal species [13,14], dietary regime [15,16], and lactation stages [17], among other factors. One study showed that the genetic variability of milk metabolites varied from 0 to 0.699 [18]. However, little information is available on the changes in milk metabolites induced by bovine β-casein variants.

A metabolomics approach is considered a powerful strategy for comprehensive metabolite analysis of milk samples. Three analytical techniques are the most widely used to investigate the milk metabolome: nuclear magnetic resonance spectroscopy (NMR), and gas and liquid chromatography-mass spectrometry (LC–MS) [19]. Among these methods, NMR can provide information on the direct relationship between metabolite abundances and resonances. LC–MS has high sensitivity and reproducibility, along with a good dynamic range, making it particularly suitable for the characterization and identification of non-volatile and high-molecular-weight metabolites [19,20,21].

In the present study, we used these metabolomics approaches to assess differences in the metabolites of bovine milk with different β-casein variants. Dairy cows were selected according to the parity, lactation stage, and health status to minimize selection bias, and metabolome profiles from milk with β-casein variants A1, A2, and heterozygote were investigated by NMR and LC–MS-based metabolomics approaches. Differences in the composition and abundance of milk metabolites among the variant groups were assessed by multivariate statistical analysis. These results can provide new insights into the differences in milk components among β-casein variants at the metabolite level and offer a potential explanation for diversity in the biological function of β-casein variants milk.

## 2. Materials and Methods 

### 2.1. Dairy Cow Selection and Milk Sample Collection

Blood samples were collected from the tail vein of 256 individual Chinese Holstein dairy cows at a local dairy farm near Chuzhou city, Anhui province, China. The experimental animal procedures were approved by the Animal Care and use Committee of the Anhui Academy of Agricultural Sciences (A11-M2-17). 

The presence of the β-casein variants A1, A2, and heterozygote A12 in each dairy cow was determined using a high-resolution melting method, as previously reported [22]. In brief, DNA was extracted from the blood samples using the Blood DNA Kit (Tiangen Biotech, Beijing, China) and amplified with a primer pair designed based on the GenBank sequence of β-casein (M16645) (F: TAAAATCCACCCCTTTGCCCA, R: CCACCACAGGGGTTTGAGTAA). The milk yield of each determined genotype of β-casein variants was recorded, and daily milk samples were used to detect somatic cell counts, and protein and fat content using the FT120 and Fossomatic 5000 systems, respectively (Foss Electric, Hillerød, Denmark). Twelve milk samples from each β-casein variant group were selected according to the criteria of a somatic cell count less than 400,000 cells/mL, and cows with parity 2–4 at lactation days 124–204. Milk yield, compositions, and somatic cell counts of β-casein variant A1A1, A2A2, and heterozygote milk are listed in Table S1. In addition, 50 mL of morning milking samples were collected and stored at −20 °C, and 10 milk samples from each variant group were applied to metabolomics analysis.

### 2.2. Nuclear Magnetic Resonance (NMR) Spectroscopy Analysis

Approximately 50 mL of milk samples from each β-casein variant group were thawed and centrifuged at 4000× *g* and 4 °C for 30 min. The top layer of the milk fat was removed, and the middle layer of skim milk (30 mL) was collected for analysis. One volume of skim milk was mixed with two volumes of methanol and one volume of MilliQ water, and the mixture was vortexed and centrifuged at 12,000× *g* and 4 °C for 10 min. The supernatant containing the metabolites was collected and dried using a freeze-drying system (Labconco Corp., Kansas City, MO, USA). Dried samples were resuspended with 600 µL of 0.1 M NaH_2_PO_4_/K_2_HPO_4_ and 0.1% NaN_3_ in MilliQ water (pH 7.4) and then centrifuged at 16,000× *g* and 4 °C for 10 min. Finally, 550 μL of the supernatant was collected from each sample and transferred into a 5-mm NMR tube for NMR spectroscopy. Milk metabolites were analyzed using an Agilent DD2600 MHz spectrometer with a proton frequency of 599.83 MHz and a temperature of 298 K (Agilent Technologies, Santa Clara, CA, USA), as described previously [23]. In brief, a one-dimensional nuclear Overhauser enhancement spectroscopy preset sequence for water resonance was applied with an acquisition time of 1.36 s. Sixty-four scans and a spectral width of 20 ppm were performed, and 32 K data points were collected. Free-induction decays of ^1^H-NMR spectra extended with a line-broadening factor of 1.0 Hz were used to enhance the signal-to-noise ratio, and then manual phasing and baseline correction were performed with MestReNova software V7.0 (Mestrelab Research, Spain). All NMR spectra were referenced to sodium trimethylsilyl-(2,2,3,3-^2^H_4_)-1-propionate and used to evaluate for the individual metabolite signals. NMR spectra were analyzed at integral regions δ 9.5–0.6 with a width of 0.002 ppm, whereas the water resonance (δ 5.20–4.72 ppm) and methanol resonance (δ 3.327–3.40 ppm) regions were removed. 

### 2.3. Liquid Chromatography Mass Spectrometry (LC–MS) Analysis

One hundred microliters of skim milk mixed with 300 µL of methanol and 10 μL of 2.9 mg/mL dl-o-chlorophenylalanine were vortexed for 30 s and centrifuged at 12,000× *g* and 4 °C for 15 min. The supernatant containing metabolites was collected for LC–MS analysis. For quality control (QC) samples, equal volumes of skim milk from all β-casein variant samples were pooled. QC samples injected around the entire samples were used to assess experimental stability.

Metabolite solutions were analyzed in both positive and negative ion modes of LC–MS (Ultimate 3000LC Orbitrap Elite; Thermo Fisher Scientific, Waltham, MA, USA). Chromatography columns equilibrated with 95% (*v/v*) buffer A (0.1% formic acid), and 4 μL solutions were loaded via an autosampler and separated on an Agilent C18 column (100 × 4.6 mm, 3 µm; Santa Clara, CA, USA) at 40 °C using buffer B (0.1% formic acid in acetonitrile) at a flow rate of 300 nL/min. The separation gradient was performed using 5–95% buffer B for 15 min. For both positive and negative ion modes, the parameters were a heater temperature of 300 °C, capillary temperature of 350 °C, sheath gas flow rate (nitrogen) of 45 arb, and auxiliary gas (nitrogen) of 15 arb. In positive ion mode, the spray voltage was set to 3.0 kV with an S-lens RF level of 30%. In negative ion mode, the spray voltage was set to 3.2 kV with an S-lens RF level of 60%. The full scan was set to 50–1000 m/z with a scan time of 0.2 s and an inter-scan time of 0.02 s. The LC–MS data were applied to feature extraction with SIEVE software (Thermo Fisher Scientific, San Jose, CA, USA). A total of 1438 features in positive-ion mode and 1631 features in negative-ion mode were obtained in this experiment.

### 2.4. Multivariate Statistical Analysis

For multivariate statistical analysis, NMR and LC–MS data were normalized and input to SIMCA-P^+^13.0 software (Umetrics AB, Umeå, Sweden). To investigate the changes in milk metabolites of the three variant groups, metabolite profiles were compared from β-casein variant A1 with those from variant A2 and heterozygote milk and those of variant A2 were compared with heterozygote milk. Unsupervised principal components analysis (PCA), supervised partial least-squares discriminant analysis (PLS-DA), and orthogonal PLS-DA (OPLS-DA) models were performed on the basis of unit variance scaling, in which the parameters R^2^X and R^2^Y were used to evaluate the model quality and confidence, and Q^2^ was applied to assess the model’s predictive ability. For the NMR data, OPLS-DA score plots were constructed to visualize the distinction between two groups, and correspondence loading plots were applied to assess the association of variables with separation of the samples on the score plots. Correlation coefficients (*r*) were calculated to assess the potential associations of metabolites in the three groups, and significant differences were considered at their cutoff *r*-value of more than 0.7. To best represent the differential metabolite signals between two groups, the colors of variable signals on the coefficient plots reflect the intensity of metabolite signals, in which red indicates a strong correlation and blue indicates no correlation. For the LC–MS data, differential metabolites were determined according to a threshold variable influence on projection (VIP) value greater than 1.0. In addition, the relative intensities of candidate metabolites were examined using an independent t-test. Metabolites with a *p*-value no more than 0.05 and a VIP value greater than 1.0 were considered to be significantly different. Signals of differential metabolites were searched against the Metabolite and Tandem MS database (https://metlin.scripps.edu/) and the human metabolome database (https://hmdb.ca/) according to the chemical shift and peak mode in NMR, and the accurate mass obtained from MS and MS/MS data with LC–MS. Pathways associated with the differential metabolites were predicted using MetaboAnalyst v.4.0 software (Xia Lab, McGill University, Montréal, Canada) [24].

## 3. Results

### 3.1. Nuclear Magnetic Resonance (NMR) Identification of Differential Milk Metabolites among β-Casein Variants 

Representative ^1^H-NMR spectra regions of δ 0.6–5.4 and 5.4–9.5 obtained from the milk samples for the three β-casein variants A1, A2, and heterozygote milk are presented in Figure S1. The unsupervised PCA of spectral signals from the milk samples for the three β-casein variants and their score plots are shown in Figure S2. Metabolome profiles demonstrated a clear tendency of separation among the three groups. Based on the supervised OPLS-DA approach, metabolite profiles between variants A1 and A2 or heterozygote A12 milk were significantly different, with Q^2^ values of at least 0.56. Score plots and coefficient loading plots of OPLS-DA were used to identify the differential metabolites between variants A1 and A2 or heterozygote A12 milk (Figure S3). However, the metabolite profiles between variants A2 and heterozygote A12 were not significantly different based on the OPLS-DA, in which samples from these two groups were not clearly separated and the Q^2^ value was less than 0. The information on differential milk metabolites from the three variants is summarized in Table 1. NMR indicated that variant A2 or heterozygote A12 milk had higher concentrations of valine, lysine, glutamate, phenylalanine, lactate, and 3-hydroxybutyrate compared to those of variant A1 milk. However, no distinct metabolites were identified between variants A2 and heterozygote A12 milk.

### 3.2. Liquid Chromatography Mass Spectrometry (LC–MS) Identification of Differential Milk Metabolites Among β-Casein Variants

Prior to data analysis, the total ion chromatograms of the QC samples were overlapped, which showed high reproducibility in the peak intensity and retention time of milk metabolite signals in both positive and negative ion modes of LC–MS (Figure S4). Unsupervised PCA was then used to assess the clustering of metabolites, followed by supervised PLS-DA and OPLS-DA, to assess the differences in milk metabolites between two groups. The Q^2^ value associated with the predictive ability of a model was at least 0.66 and 0.55 in positive and negative ion modes, respectively.

**Figure 1:**
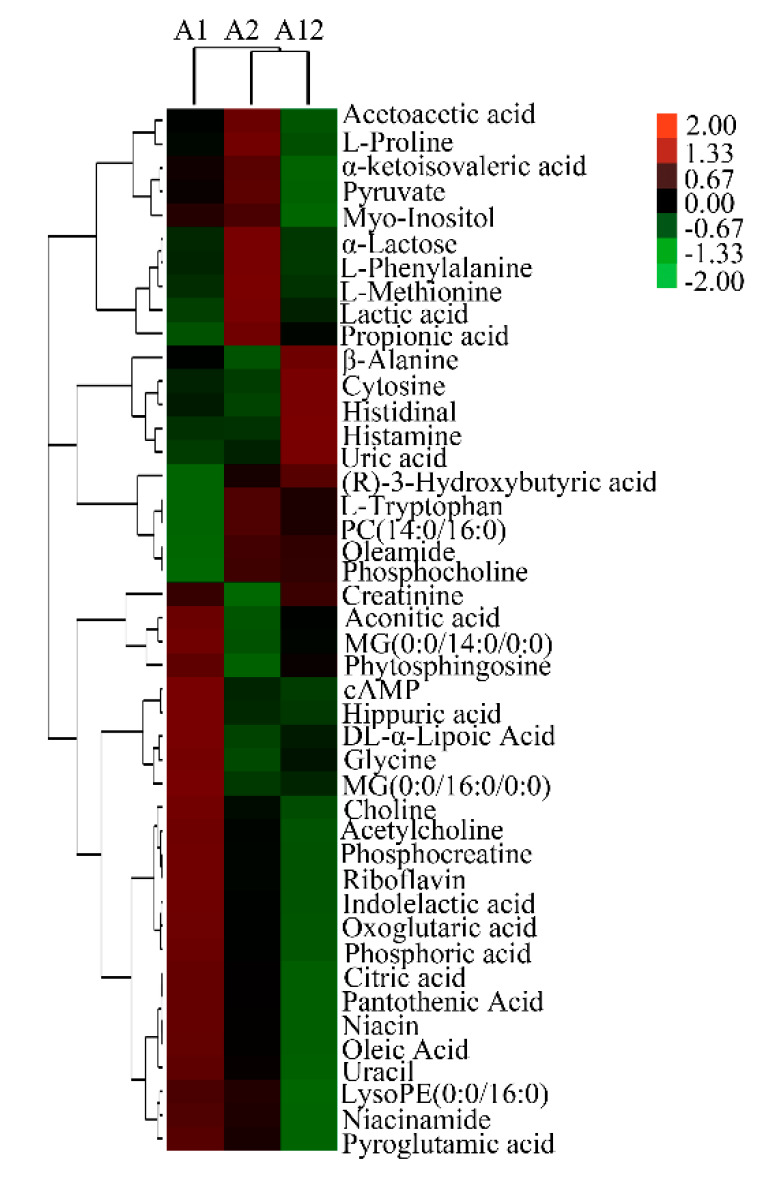
Hierarchical clustering of differential metabolites from β-casein variants A1/A1 (A1), A2/A2 (A2), and heterozygote A1/A2 (A12) milk. PC(14:0/16:0): Phosphatidylcholines (14:0/16:0); MG(0:0/16:0/0:0): Monoradyglycerols (0:0/16:0/0:0); cAMP: cyclic adenosine monophosphate. 3.3. Metabolic Pathways of Differential Metabolites Among β-Casein Variants.

Several of the identified milk metabolites showed differential abundance between variants A1 and A2, with choline, phytosphingosine, and cyclic adenosine monophosphate (cAMP) detected at significantly higher levels, whereas oleamide, methionine, and phenylalanine were detected at significantly lower levels in variant A1. Similarly, several metabolites, such as choline, acetylcholine, and cAMP, were found at significantly lower abundance, whereas oleamide, uric acid, and cytosine were significantly more abundant in heterozygote A12 than variant A1 milk. Methionine, lysoPE (0:0/16:0), and myo-inositol were significantly less abundant, whereas uric acid, phosphocholine, tryptophan, and cytosine were significantly more abundant in heterozygote A12 than variant A2 milk. Collectively, metabolites of choline and cAMP were most abundant in variant A1 milk and methionine and proline were most abundant in variant A2 milk. The identified differential metabolites presented by hierarchical clustering are listed in Figure 1, and the information of differential metabolites with VIP values > 1.0 and *p*-values < 0.05 obtained from positive and negative ion mode are summarized in Tables S2 and S3, respectively.

**Figure 2:**
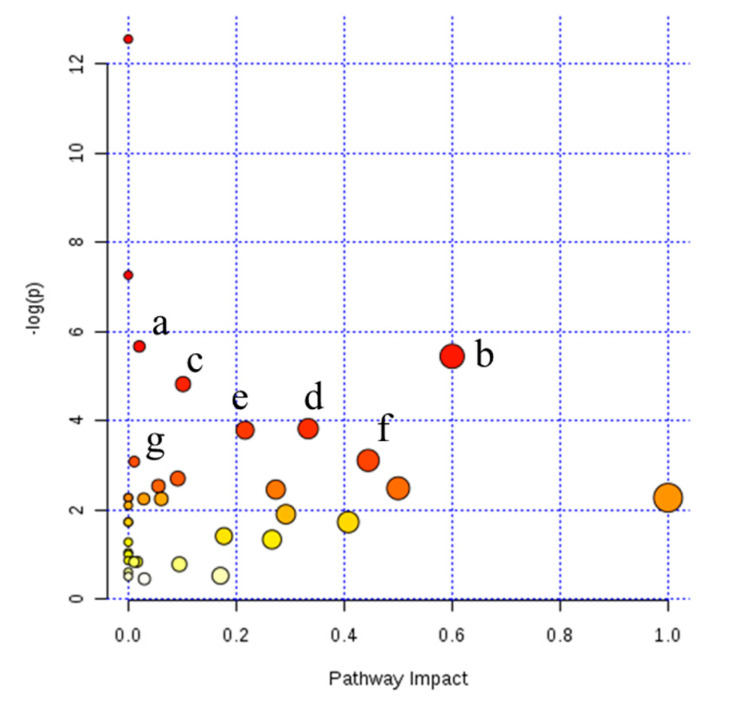
Pathway analysis of differential metabolites between β-casein variants A1/A1 and A2/A2 milk with MetaboAnalyst analysis. (a) Pantothenate and coenzyme A biosynthesis; (b) synthesis and degradation of ketone bodies; (c) butanoate metabolism; (d) valine, leucine and isoleucine biosynthesis; (e) glycerophospholipid metabolism; (f) glyoxylate and dicarboxylate metabolism; (g) valine, leucine and isoleucine degradation. Pathway impact on the horizontal axis calculated from pathway topology analysis; −log(*p*) on the vertical axis calculated from the pathway enrichment analysis represented by the negative logarithm transformation of *p*-value.

The pathways associated with the differential milk metabolites identified from NMR and LC–MS were predicted by MetaboAnalyst software (Xia Lab, McGill University, Montréal, Canada). The differential milk metabolites between variants A1 and A2 were assigned to pantothenate and coenzyme A (CoA) biosynthesis, synthesis and degradation of ketone bodies, butanoate metabolism, and valine, leucine, and isoleucine biosynthesis (Figure 2, Table S3). Differential milk metabolites between variants A1 and heterozygote A12 were assigned to pantothenate and CoA biosynthesis; d-glutamine and d-glutamate metabolism; alanine, aspartate, and glutamate metabolism; glutathione metabolism (Figure S5). Differential milk metabolites between A2 and heterozygote A12 were involved in butanoate metabolism, β-alanine metabolism, citrate cycle, and pyruvate metabolism (Figure S6).

## 4. Discussion

Several previous studies have shown that genetic variants of β-casein were significantly associated with major milk proteins, including α_S1_-casein, β-casein, κ-casein, and β-lactoglobulin [8,9]. Additionally, β-casein variants A1 and A2 have been shown to contribute to a difference in casein micelle assembly and molecular chaperone activity [2]. These results suggested that β-casein variants can influence milk components, even from only a part of the protein fraction. The present study further expands upon these previous findings by providing the first assessment of metabolite profiles from β-casein variants A1, A2, and heterozygote milk using NMR- and LC-MS-based metabolomics approaches. Differential metabolites among the β-casein variant groups were clearly identified. Moreover, some of the distinct metabolites between variants A2 and A12 identified by the LC–MS approach were not obtained by the NMR technique coupled with multivariate statistical analysis. This demonstrates the higher sensitivity of LC–MS than NMR in metabolomics analysis, as widely reported previously [19,21]. Given that several milk metabolites were uniquely identified in β-casein variants milk using NMR- and LC-MS-based metabolomics approaches, integrating these two approaches is a preferred method to obtain a deeper metabolome characterization [25].

Using both NMR and LC-MS metabolomics approaches, we found that the relative abundances of methionine, proline, acetoacetic acid, and α-lactose were significantly higher in the β-casein variant A2 milk than in either variant A1 or heterozygote A12 milk. By contrast, the levels of choline, glycine, citric acid, and cAMP were significantly higher in variant A1 milk. These results, thus, extend knowledge of the components of milk corresponding to β-casein variants at the metabolomics level, which can help to further characterize differences in the physiological functionality of variants A1 and A2 milk. In general, methionine and lysine have been considered as the central limiting factors for milk protein synthesis in the mammary gland of dairy cows. Although the reported effects of methionine supplementation on the concentration of milk proteins in lactating dairy cows are inconsistent, many previous studies have suggested that methionine addition to the diet tends to increase the protein yield and methionine content in the blood [26,27,28]. Cows with the β-casein variant A2 also tend to have a higher milk yield than those with variant A1 and show increased breeding values for milk and protein yield [29,30]. Thus, we suggest that the increased metabolite of methionine may be associated with the observed higher protein yield in variant A2 milk. Proline and hydroxyproline are also among the most abundant components in milk proteins [31], and supplementation of proline improves the efficiency of dietary energy utilization in dairy cows [32]. Besides playing important roles in protein synthesis, these metabolites are also involved in multiple biochemical and physiological processes such as increasing antioxidative activities and immune responses [33,34]. However, little is known about the physiological functions of high methionine and proline concentrations in variant A2 milk. Thus, further studies are required to clarify the associations of these amino acids with β-casein variants and their biological functions.

Glycine, choline, and cAMP were the most abundant metabolites identified in β-casein variant A1 milk. In addition to its main function as an activator of the mammalian target of rapamycin signaling pathway for protein biosynthesis [35], glycine also participates in the biosynthesis of glutathione, nucleic acids, and uric acid [36]. In particular, glutathione is one of the primary antioxidant agents in cells [37]. Choline has been recognized as an essential nutrient [38]. Choline concentration in human mature milk was shown to be stable regardless of maternal age and intake [39]. However, limited data are available on choline concentration in bovine milk related to β-casein variants. Since methionine showed the highest abundance in variant A2 milk, we questioned whether or not the high choline content in variant A1 milk may have crucial biological function, because S-adenosyl-l-methionine—a metabolite of methionine-serves as a methyl donor for choline biosynthesis [40]. Importantly, choline acts as a precursor for the biosynthesis of acetylcholine (a major transmitter for nerve and leukocyte function), and various phospholipids, which are essential components of the cellular membranes, and further contribute to modulating macrophage interleukin (IL)-1β and IL-18 production [38,41]. Therefore, the physiological function of choline in variant A1 milk warrants further investigation. cAMP, a derivative of adenosine triphosphate (ATP), is a secondary messenger that widely contributes to intracellular signal transduction based on cAMP-dependent pathways. A study of the cAMP concentration in mammary glands and milk throughout the lactation cycle in the guinea pig indicated that cAMP could inhibit lactose synthesis [42]. As discussed previously, we speculated that a higher concentration of cAMP in variant A1 milk may be related to the lower concentration of α-lactose observed in our study. However, further research is required to establish any causal relationships and mechanisms among the milk metabolites of β-casein variant milk.

Cytosine and uric acid were the most abundant metabolites identified in heterozygote A12 milk. Cytosine, as one of the four main bases, is considered to be a microbial marker in the rumen [43] and is also detected in the milk of humans and dairy animals [44]. Thus, we suggest that the identified cytosine originating from the rumen may transfer into the blood, which is then taken up by the mammary gland to contribute to protein biosynthesis. However, sparse information is available concerning the relationship of the cytosine with β-casein variant milk. Uric acid, a metabolite of nucleotides, has also been reported to be highly abundant in milk and related to breeding and diet nutrition [16,45]. Unexpectedly, glycine, a precursor of uric acid, had the highest abundance in A1 milk, as discussed earlier. Therefore, the large amount of uric acid in heterozygote A12 milk could result from the degradation of nucleic acids or from biosynthesis and secretion in response to the nutritional demand of offspring. These nucleotides play crucial roles in nucleic acids biosynthesis and biochemical pathways, and several previous studies have suggested that nucleotides in formula or milk have an immunomodulatory role in neonates [12,46]. However, the mechanisms contributing to the higher concentrations of several nucleotides in heterozygote A12 milk remain to be clarified with further investigation.

## 5. Conclusions

In summary, the effect of β-casein variants on the metabolite components of bovine milk were revealed using NMR- and LC-MS-based metabolomics approaches. Several metabolites such as methionine, proline, and α-lactose were found to be more abundant in β-casein variant A2 milk, whereas choline, glycine, citric acid, and cAMP were more abundant in variant A1 milk. These findings provide novel insights into the differences in milk components among β-casein variants at the metabolite level. Moreover, these data offer useful resource for further research on the physiological implications of β-casein variants. In particular, further studies are needed to discriminate among the several factors influencing milk components, such as diet, environment, and physiological status.

## Figures and Tables

**Table 1:** Differential metabolites from β-casein variants A1/A1 (A1) and A2/A2 (A2) or heterozygote A1/A2 (A12) milk.

Metabolites	Chemical Shift ^a^	Correlation Coefficients	log2(Fold -Change)	*p*-Value
heterozygote A1/A2 comparative with variant A1/A1				
Valine	1.04 (d), 1.00 (d)	0.841	0.45	9.82 × 10^−5^
3-hydroxybutytrate	1.20 (d)	0.836	0.57	6.73 × 10^−5^
Lactate	1.33 (d),4.12 (q)	0.826	0.41	8.44 × 10^−5^
Alanine	1.48 (d)	0.785	0.49	3.08 × 10^−4^
Lysine	1.74 (m),1.90 (m)	0.773	0.43	4.53 × 10^−4^
Acetate	1.92 (s)	0.831	0.48	9.84 × 10^−5^
Glutamate	2.07 (m),2.35 (m)	0.739	0.43	1.05 × 10^−3^
Phenylalanine	7.24 (m),7.30 (m),7.36 (m)	0.88	0.68	1.69 × 10^−6^
Histidine	7.08 (s),7.85 (s)	0.891	0.57	5.07 × 10^−4^
Unknown metabolite	6.20 (s)	0.710	0.60	0.0019
variant A2/A2 comparative with A1/A1				
Valine	1.04 (d), 1.00 (d)	0.791	0.41	3.59 × 10^−4^
3-hydroxybutytrate	1.20 (d)	0.792	0.46	6.70 × 10^−4^
Lactate	1.33 (d),4.12 (q)	0.844	0.39	6.45 × 10^−5^
Alanine	1.48 (d)	0.751	0.42	1.09 × 10^−3^
Lysine	1.74 (m),1.90 (m)	0.717	0.37	1.76 × 10^−3^
Acetate	1.92 (s)	0.764	0.40	6.70 × 10^−4^
Glutamate	2.07 (m),2.35 (m)	0.733	0.41	1.42 × 10^−3^
Phenylalanine	7.24 (m),7.30 (m),7.36 (m)	0.693	0.57	1.27 × 10^−3^
Histidine	7.08 (s),7.85 (s)	0.734	0.41	1.94 × 10^−3^

^a^ Multiplicity: s, singlet; d, doublet; t, triplet; q, quartet; m, multiplet.

## Supplementary Materials

The following are available online at https://www.mdpi.com/2076-2615/10/6/954/s1, Figure S1: Representative 1H-NMR spectra region of δ 0.6–5.4 and 5.4–9.5 obtained from β-casein variants A1/A1 (A1), A2/A2 (A2), and heterozygote A1/A2 (A12) milk, Figure S2: An overview of 3D principal component analysis score plots of nuclear magnetic resonance spectra signals from β-casein variants A1/A1 (A1), A2/A2 (A2), and heterozygote A1/A2 (A12) milk, Figure S3: The score plots and coefficient loading plots of orthogonal partial least squares discriminant analysis from nuclear magnetic resonance spectral signals between variants A1/A1 (A1), A2/A2 (A2), and heterozygote A1/A2 (A12) milk, Figure S4: The total ion chromatogram of QC samples from all β-casein variants A1/A1 (A1), A2/A2 (A2), and heterozygote A1/A2 (A12) milk in positive-mode (a) and negative-ion mode (b) of LC–MS, Figure S5: Pathway analysis of differential metabolites between variant A1/A1 and heterozygote milk with MetaboAnalyst software, Figure S6: Pathway analysis of differential metabolites between variant A2/A2 and heterozygote milk with MetaboAnalyst, Table S1: The information of milk samples among bovine β-casein variant A1/A1, A2/A2, and heterozygote milk, Table S2: The differential metabolites from β-casein variants A1/A1 and A2/A2 or heterozygote milk in positive ion mode of LC–MS data, Table S3: The differential metabolites from β-casein variants A1/A1 and A2/A2 or heterozygote milk in negative ion mode of LC–MS data, Table S4: The metabolic pathways of differential metabolites from variants A1/A1 and A2/A2 milk with P values no greater than 0.5.
