# Supplementary material for: Changes in Metabolites from Bovine Milk with β-Casein Variants Revealed by Metabolomics
Source: Animals (Basel). 2020 May 30;10(6):954. doi: 10.3390/ani10060954 (PMC7341322; doi:10.3390/ani10060954)
Supplement: Supplementary file 1 [file animals-10-00954-s001.pdf]

Table S1. The information of milk samples among bovine β-casein variant A1/A1, A2/A2, and heterozygote milk.

Terms	Variant A1/A1 (12 cows)	Variant A2/A2 (12 cows)	Heterozygote (12 cows)	*p*-value
Milk yield, Kg/d	30.3±4.1	31.1±5.1	31.4±5.6	0.843*
Fat, %	3.56±0.31	3.61±0.35	3.69±0.30	0.583*
Protein, %	3.27±0.26	3.21±0.15	3.23±0.17	0.713*
Somatic cell counts, 10^4 cell /mL	15.3±13.1	12.2±10.6	15.4±12.8	0.767*
Lactation, d #	166(129-204)	148(124-179)	149(128-194)	

* data analyzed by one way ANOVA followed by Tukey test. # median and range.

Table S2. The differential metabolites from β-casein variants A1/A1 and A2/A2 or heterozygote milk in positive ion mode of LC–MS data.

Metabolites name	CompMW	Δppm	Time	MZ	VIP	*p* value	Log2(Fold-change)
variant A1/A1 comparative with A2/A2							
Oleamide	281.2727	2	9.9676	282.2799	2.2583	1.29E-04	-2.97
L-Methionine	149.0513	1	1.1774	150.0586	1.7531	7.62E-03	-0.59
PC(14:0/16:0)	705.5343	4	9.7783	706.5416	1.6010	1.71E-02	-0.58
L-Phenylalanine	165.0796	3	1.9010	166.0869	1.4740	3.06E-02	-0.34
L-Proline	115.0636	2	0.8825	99.0443	2.2776	1.03E-04	-0.26
Acetoacetic acid	102.0318	1	1.1716	103.0391	2.0993	6.42E-04	-0.21
α-ketoisovaleric acid	116.0477	3	1.1854	117.0549	1.3670	4.75E-02	-0.12
Creatinine	113.0591	1	1.0261	114.0664	1.3787	4.53E-02	0.14
L-Tryptophan	204.0868	15	0.7397	103.0507	1.5384	2.30E-02	0.19
Phosphocholine	183.0681	11	0.7350	92.5413	1.5437	2.24E-02	0.21
Aconitic acid	174.0171	3	1.1793	175.0244	1.3576	4.92E-02	0.25
Hippuric acid	179.0589	3	3.6774	180.0661	1.4092	4.01E-02	0.34
Glycine	75.0320	0	3.8275	76.0393	1.4270	3.73E-02	0.42
Uric acid	168.0277	3	1.1757	210.0615	1.8359	4.62E-03	0.48
MG(0:0/16:0/0:0)	330.2782	3	9.8902	331.2855	1.4081	4.03E-02	0.52
MG(0:0/14:0/0:0)	302.2466	2	8.7547	303.2539	1.4891	2.87E-02	0.58
cAMP	329.0538	3	1.1836	330.0611	1.7308	8.65E-03	0.84
Phytosphingosine	317.2939	2	5.6445	318.3012	1.4944	2.80E-02	1.24
Choline	103.0999	1	7.9097	104.1072	1.7619	7.24E-03	1.97
variant A1/A1 comparative with heterozygote							
Oleamide	281.2719	2	9.9676	282.2799	1.2029	4.39E-03	-2.67
Uric acid	168.0283	3	1.1757	210.0615	1.0204	1.97E-02	-0.90
Cytosine	111.0433	1	12.6408	112.0507	1.5390	5.21E-05	-0.63
Histidinal	139.0746	2	15.8338	140.0821	1.0493	1.60E-02	-0.40
Histamine	111.0796	1	15.6717	112.0871	1.0992	1.09E-02	-0.32
Niacin	123.032	15	0.7552	62.5242	1.1800	5.43E-03	0.22
Citric acid	192.027	5	1.1778	215.0172	1.2145	3.93E-03	0.40
Phosphoric acid	97.9769	1	1.2621	98.9843	1.2966	1.68E-03	0.41
Niacinamide	122.048	3	1.1770	123.0558	1.0049	2.20E-02	0.45
Oxoglutaric acid	146.0215	2	1.1799	129.0186	1.5773	2.46E-05	0.47
Pantothenic Acid	219.1107	3	2.2083	242.1008	1.3181	1.31E-03	0.47
Pyroglutamic acid	129.0426	1	1.1799	113.0235	1.5879	1.97E-05	0.55
Riboflavin	376.1383	2	3.6435	377.1467	1.2752	2.12E-03	0.55

Uracil	112.0273	2	1.1802	113.0349	1.5185	7.54E-05	0.66
Indolelactic acid	205.0739	3	4.1420	206.0820	1.0258	1.90E-02	0.66
Glycine	75.032	0	3.8275	76.0393	1.2969	1.67E-03	0.68
Phosphocreatine	211.0358	3	1.1826	212.0438	1.0655	1.41E-02	0.73
cAMP	329.0525	3	1.1836	330.0611	1.3111	1.42E-03	1.00
Acetylcholine	145.1103	2	0.8787	184.0739	1.3661	7.34E-04	1.15
Choline	103.0997	1	7.9097	104.1072	1.3572	8.22E-04	2.74
variant A2/A2 comparative with heterozygote							
Uric acid	168.0277	3	1.1757	210.0615	1.2741	7.64E-03	-1.38
Phosphocholine	183.0681	11	0.7350	92.5413	1.0089	4.37E-02	-0.82
L-Tryptophan	204.0868	15	0.7397	103.0507	1.0027	4.52E-02	-0.75
Cytosine	111.0434	1	12.6408	112.0507	1.6111	2.06E-04	-0.64
Histidinal	139.0749	2	15.8338	140.0821	1.4118	2.26E-03	-0.51
Histamine	111.0798	1	15.6717	112.0871	1.1790	1.54E-02	-0.29
β-Alanine	89.0478	1	0.8695	90.0551	1.0547	3.38E-02	-0.21
Oxoglutaric acid	146.0219	2	1.1799	129.0186	1.2976	6.32E-03	0.31
L-Proline	115.0636	2	0.8825	99.0443	1.2322	1.05E-02	0.40
Pyroglutamic acid	129.0428	1	1.1799	113.0235	1.6717	7.89E-05	0.47
Uracil	112.0276	2	1.1802	113.0349	1.3790	3.10E-03	0.49
LysoPE(0:0/16:0)	453.2872	3	7.4512	454.2944	1.0941	2.67E-02	0.52
L-Methionine	149.0513	1	1.1774	150.0586	1.1287	2.15E-02	0.61

Table S3. The differential metabolites from β-casein variants A1/A1 and A2/A2 or heterozygote milk in negative ion mode of LC–MS data.

Metabolites name	CompMW	Δppm	Time	MZ	VIP	*p* value	Log2(Fold-change)
variant A1/A1 comparative with A2/A2							
Propionic acid	74.0373	7	0.8208	73.0300	1.4160	1.59E-02	-0.28
Alpha-Lactose	342.1165	0	1.0778	387.1147	1.3712	2.03E-02	-0.13
DL-α-Lipoid acid	206.0429	3	1.6090	205.0357	1.2495	3.75E-02	0.13
Hippuric acid	179.0587	2	3.6820	178.0514	1.2939	3.03E-02	0.29
Aconitic acid	174.0198	19	1.1750	173.0125	1.2382	3.95E-02	0.50
Citric acid	192.0307	19	1.1497	191.0234	1.2144	4.41E-02	0.52
Glycine	75.0324	4	3.8274	74.0251	1.5071	9.19E-03	0.64
Pantothenic acid	219.1150	19	2.2052	218.1077	1.4549	1.27E-02	0.74
cAMP	329.0533	2	1.1992	328.0460	1.3035	2.89E-02	0.75
variant A1/A1 comparative with heterozygote							
(R)-3-Hydroxybutyric acid	104.0477	3	1.3069	103.0405	1.5880	9.78E-03	-0.30
DL-α-Lipoid acid	206.0429	3	1.6090	205.0357	1.4737	1.82E-02	0.10
Riboflavin	376.1386	0	3.6406	375.1313	1.4560	1.98E-02	0.41
Pantothenic acid	219.1150	19	2.2052	218.1077	1.2950	4.18E-02	0.44
Oleic acid	282.2557	0	8.1743	281.2484	1.9230	9.35E-04	0.59
Indolelactic acid	205.0742	1	4.1476	204.0669	1.2961	4.16E-02	0.60
cAMP	329.0533	2	1.1992	328.0460	1.6706	5.95E-03	0.82
variant A2/A2 comparative with heterozygote							
Alpha-Lactose	342.1165	0	1.0778	387.1147	1.8828	1.61E-02	0.19
Pyruvate	88.0161	0	1.1697	87.0088	1.8688	1.70E-02	0.09
Acetoacetic acid	102.0320	2	1.0311	101.0247	1.8372	1.94E-02	0.23
Lactic acid	90.0315	2	1.1517	89.0243	1.6363	4.10E-02	0.27
myo-Inositol	180.0620	7	1.0771	179.0547	1.8119	2.15E-02	0.91

Table S4. The metabolic pathways of differential metabolites from variants A1/A1 and A2/A2 milk with P values no greater than 0.5.

Pathway name	Total	Expected	Hits	-Log (Raw P)	Impact
Pantothenate and CoA biosynthesis	15	0.324	3	5.666	0.020
Synthesis and degradation of ketone bodies	5	0.108	2	5.444	0.600
Butanoate metabolism	20	0.431	3	4.821	0.101
Valine, leucine and isoleucine biosynthesis	11	0.237	2	3.820	0.333
Glycerophospholipid metabolism	29	0.625	3	3.787	0.216
Glyoxylate and dicarboxylate metabolism	16	0.345	2	3.107	0.444
Valine, leucine and isoleucine degradation	38	0.820	3	3.080	0.011

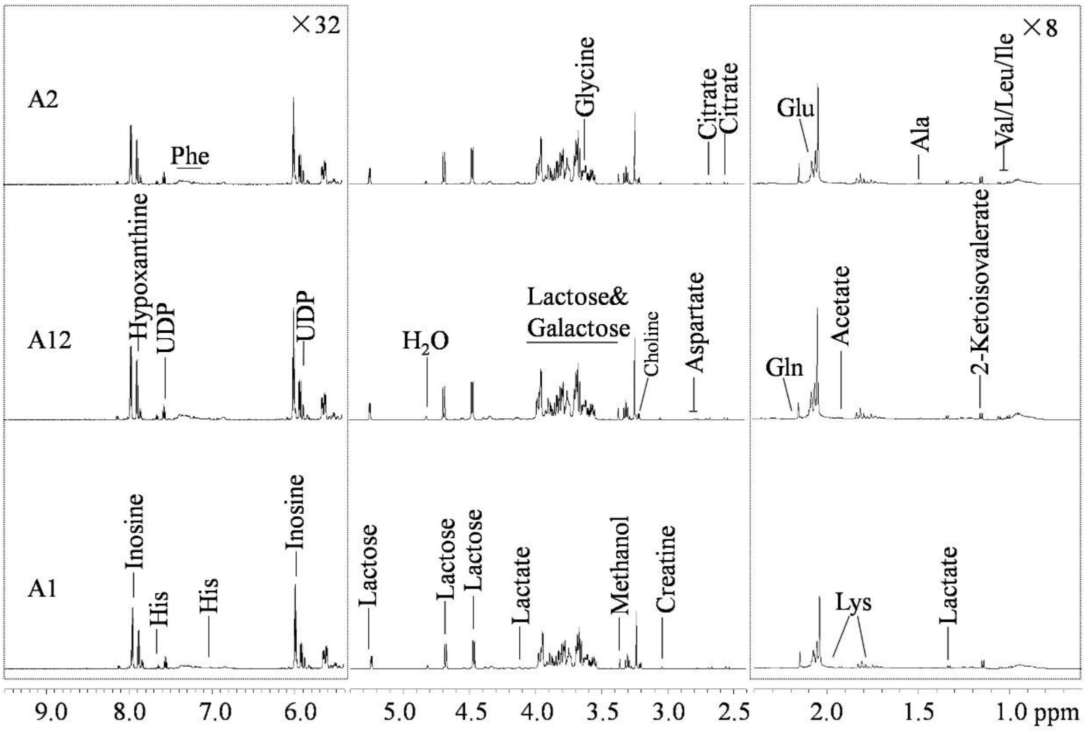

Figure S1. Representative ^{1}H-NMR spectra region of δ 0.6–5.4 and 5.4–9.5 obtained from β-casein variants A1/A1 (A1), A2/A2 (A2) and heterozygote (A12) milk. The regions of δ 0.6–5.4 and 5.4-9.5 were extended 8 and 32 times, respectively, compared with spectra region of δ 2.4–5.4. Ala, Alanine; Val, Valine; Ile, Isoleucine; Leu, Leucine; Glu, Glutamate; Phe, Phenylalanine; UDP, Uridine diphosphate; Gln, Glutamine; Lys, Lysine; His, Histidine.

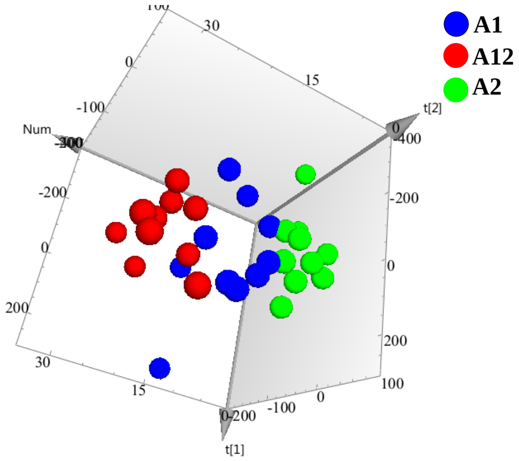

R2X[1] = 0.754 R2X[2] = 0.121

Figure S2. An overview of 3D principal component analysis score plots of nuclear magnetic resonance spectra signals from β-casein variants A1/A1 (A1), A2/A2 (A2) and heterozygote A1/A2 (A12) milk.

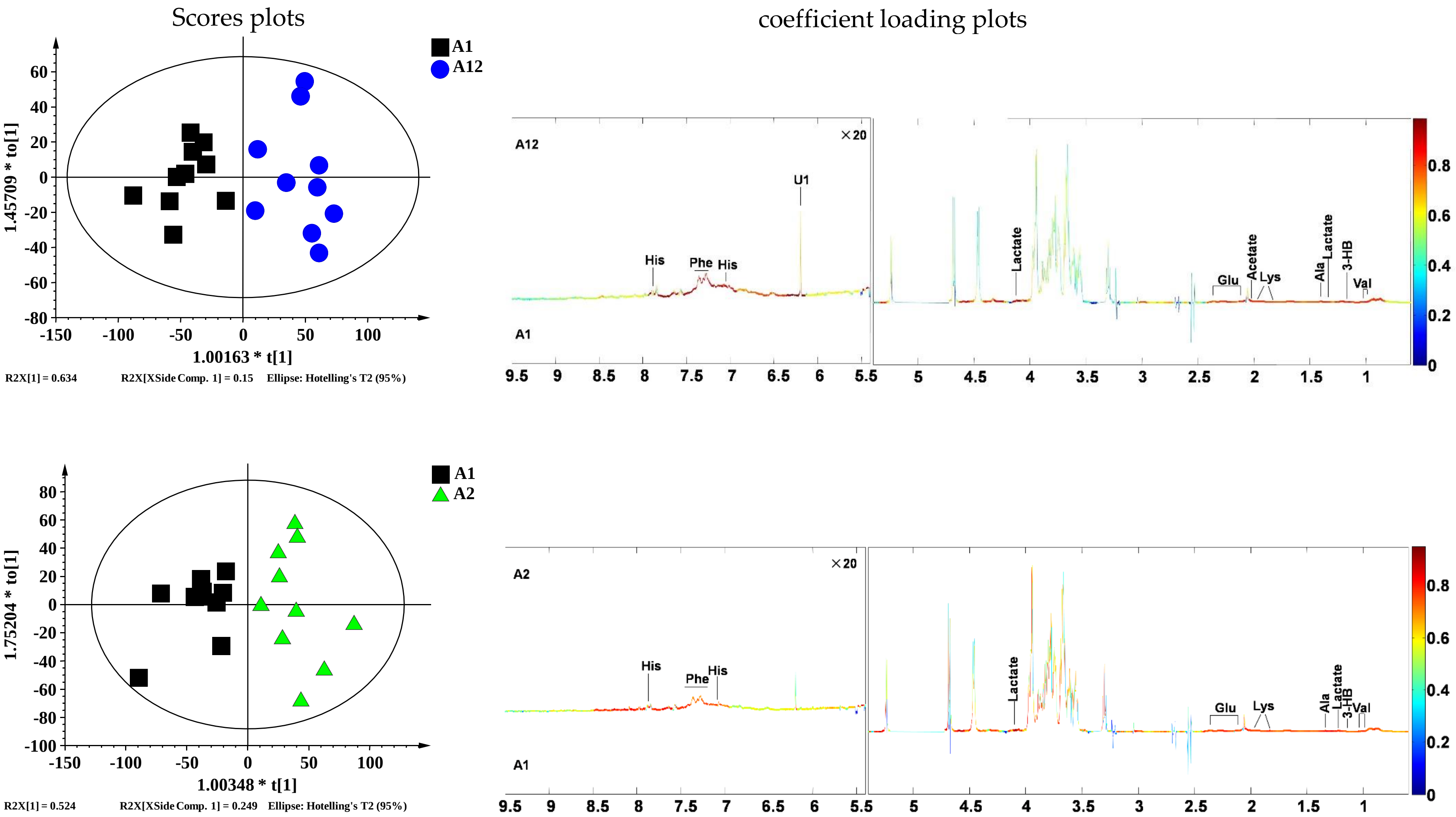
Scores plots
coefficient loading plots
A1
A12
1.45709 * to[1]
1.00163 * t[1]
R2X[1] = 0.634
R2X[XSide Comp. 1] = 0.15
Ellipse: Hotelling's T2 (95%)
A12
A1
×20
U1
His
Phe
His
Lactate
Glu
Acetate
Lys
Ala
Lactate
3-HB
Val
A1
A2
1.75204 * to[1]
1.00348 * t[1]
R2X[1] = 0.524
R2X[XSide Comp. 1] = 0.249
Ellipse: Hotelling's T2 (95%)
A2
A1
×20
His
Phe
His
Lactate
Glu
Lys
Ala
Lactate
3-HB
Val

Figure S3. The score plots and coefficient loading plots of orthogonal partial least squares discriminant analysis from nuclear magnetic resonance spectral signals between variants A1/A1 (A1), A2/A2 (A2) and heterozygote A1/A2 (A12) milk. Ala, Alanine; Val, Valine; Ile, Isoleucine; Leu, Leucine; Glu, Glutamate; Phe, Phenylalanine; 3-HB, 3-Hydroxybutyrate; Lys, Lysine; His, Histidine; U1, unknown metabolite.

a

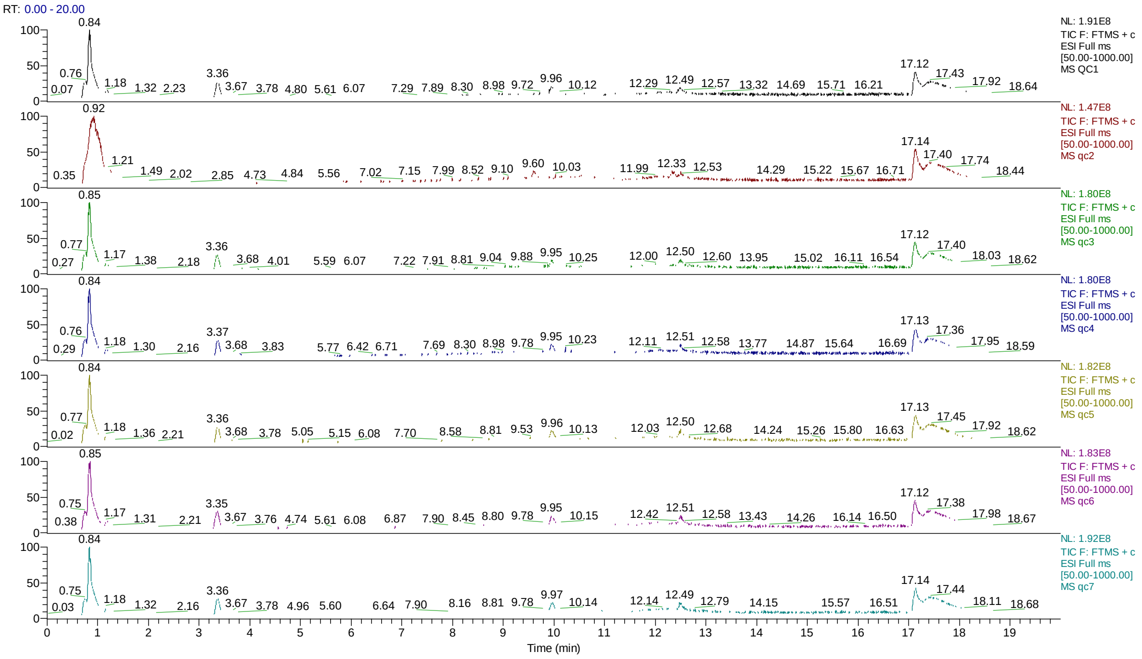

b

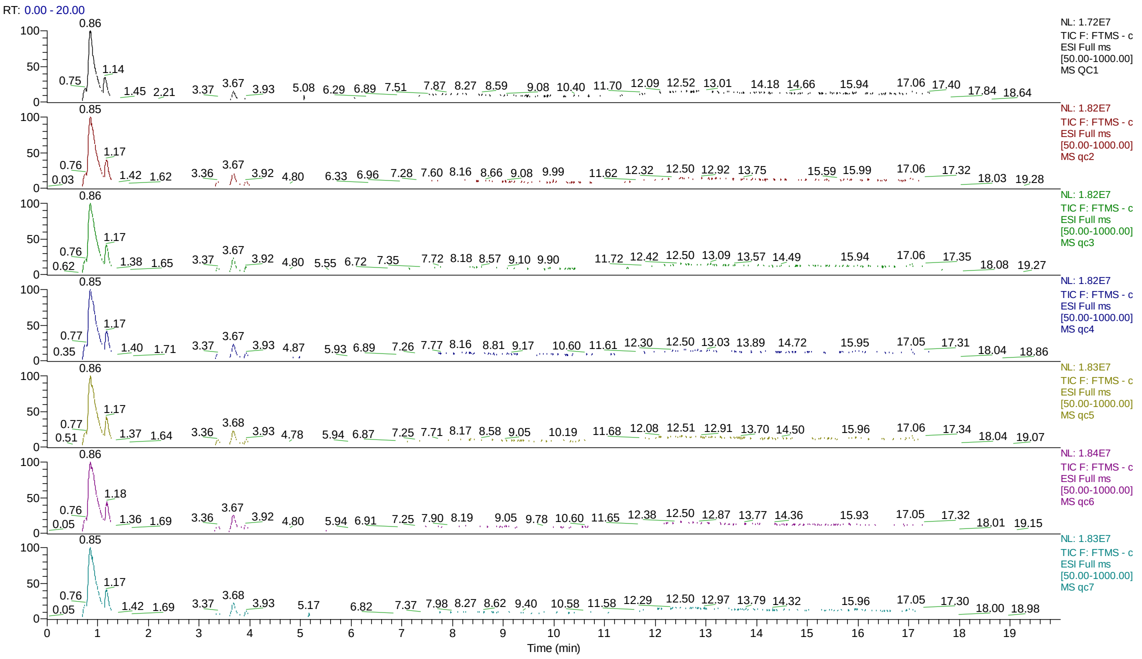

Figure S4. The total ion chromatogram of QC samples from all β-casein variants A1/A1 (A1), A2/A2 (A2) and heterozygote A1/A2 (A12) milk in positive-mode (a) and negative-ion mode (b) of LC-MS.

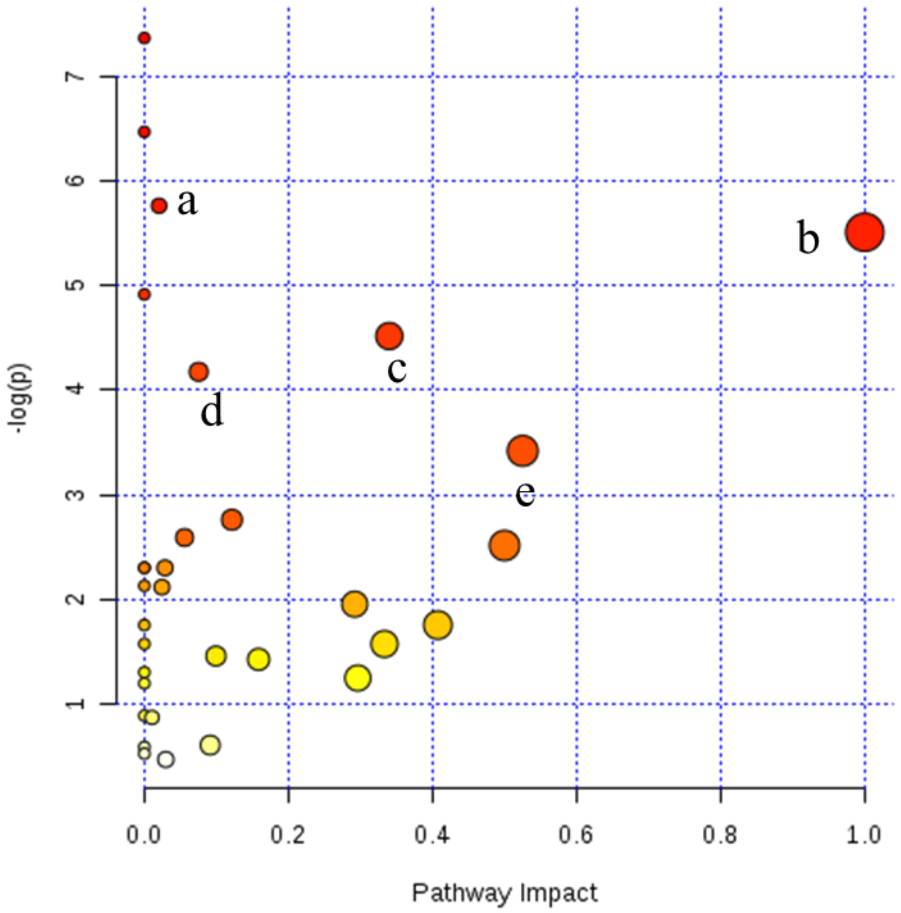

Figure S5. Pathway analysis of differential metabolites between variant A1/A1 and heterozygote milk with MetaboAnalyst software. (a) Pantothenate and CoA biosynthesis; (b) D-Glutamine and D-glutamate metabolism; (c) Alanine, aspartate and glutamate metabolism; (d) Glutathione metabolism; (e) Histidine metabolism. Pathway impact on the horizontal axis calculated from pathway topology analysis; -log(p) on the vertical axis calculated from the pathway enrichment analysis represented by the negative logarithm transformation of p value.

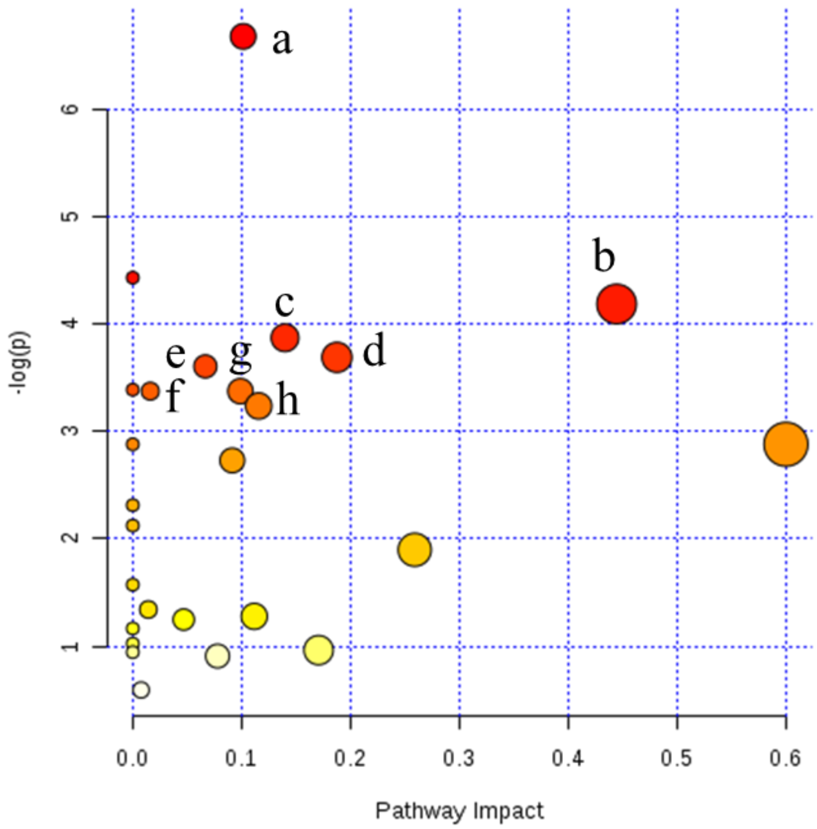

Figure S6. Pathway analysis of differential metabolites between variant A2/A2 and heterozygote milk with MetaboAnalyst. (a) Butanoate metabolism; (b) beta-Alanine metabolism; (c) Citrate cycle; (d) Pyruvate metabolism; (e) Alanine, aspartate and glutamate metabolism; (f) Galactose metabolism; (g) Glycolysis or Gluconeogenesis; (h) Cysteine and methionine metabolism. Pathway impact on the horizontal axis calculated from pathway topology analysis; -log(p) on the vertical axis calculated from the pathway enrichment analysis represented by the negative logarithm transformation of p value.
